# Supplementary material for: Splice-Junction-Based Mapping of Alternative Isoforms in the Human Proteome
Source: Cell Rep. Author manuscript; Available in PMC 2020 Jan 15. (PMC6961840; doi:10.1016/j.celrep.2019.11.026)
Supplement: 2 [file NIHMS1546469-supplement-2.zip › DF1/PXD010154/Lung/Lung_1_EEF1D_FEEHVQSVDIAAFNKL.pdf]

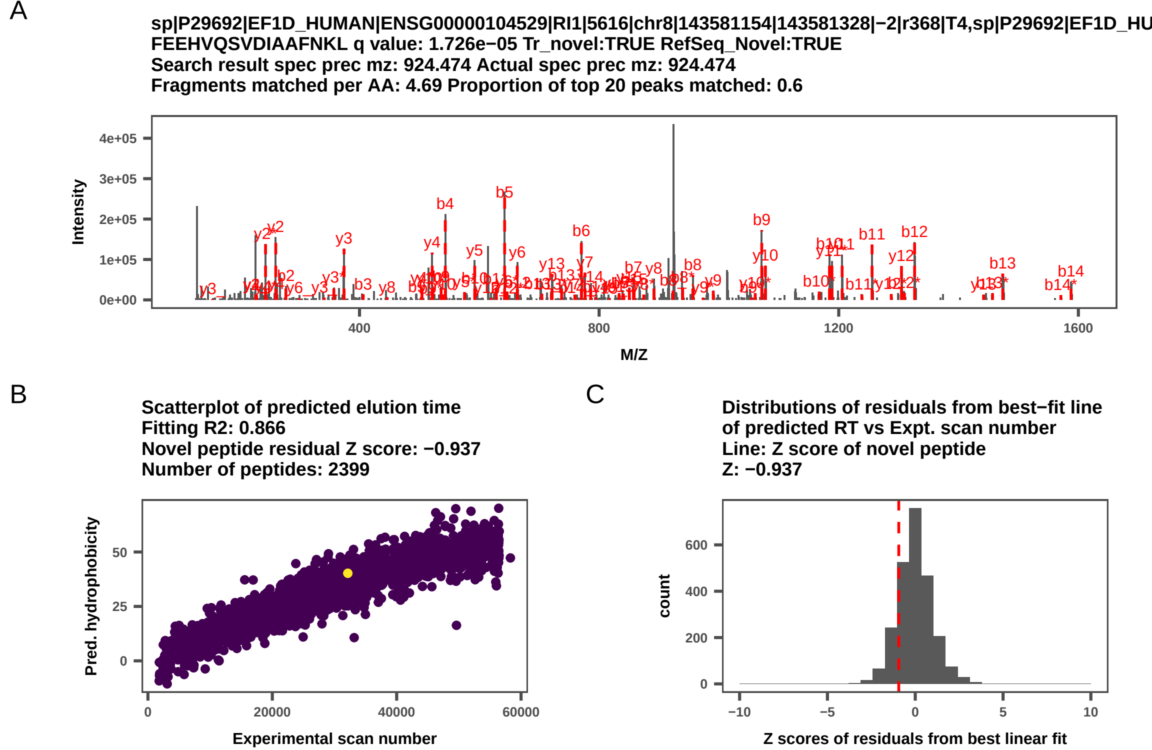
A
sp|P29692|EF1D_HUMAN|ENSG00000104529|RI1|5616|chr8|143581154|143581328|−2|r368|T4,sp|P29692|EF1D_HU
FEEHVQSVDIAAFNKL q value: 1.726e−05 Tr_novel:TRUE RefSeq_Novel:TRUE
Search result spec prec mz: 924.474 Actual spec prec mz: 924.474
Fragments matched per AA: 4.69 Proportion of top 20 peaks matched: 0.6
Intensity
M/Z
B
Scatterplot of predicted elution time
Fitting R2: 0.866
Novel peptide residual Z score: −0.937
Number of peptides: 2399
Pred. hydrophobicity
Experimental scan number
C
Distributions of residuals from best−fit line
of predicted RT vs Expt. scan number
Line: Z score of novel peptide
Z: −0.937
count
Z scores of residuals from best linear fit
